# Supplementary material for: Integrating WGCNA, TCN, and Alternative Splicing to Map Early Caste Programs in Day-2 Honeybee Larvae
Source: Genes (Basel). 2025 Nov 26;16(12):1409. doi: 10.3390/genes16121409 (PMC12733025; doi:10.3390/genes16121409)
Supplement: Supplementary file 1 [file genes-16-01409-s001.zip › Supplemental/Supplemental Figure 2.pdf]

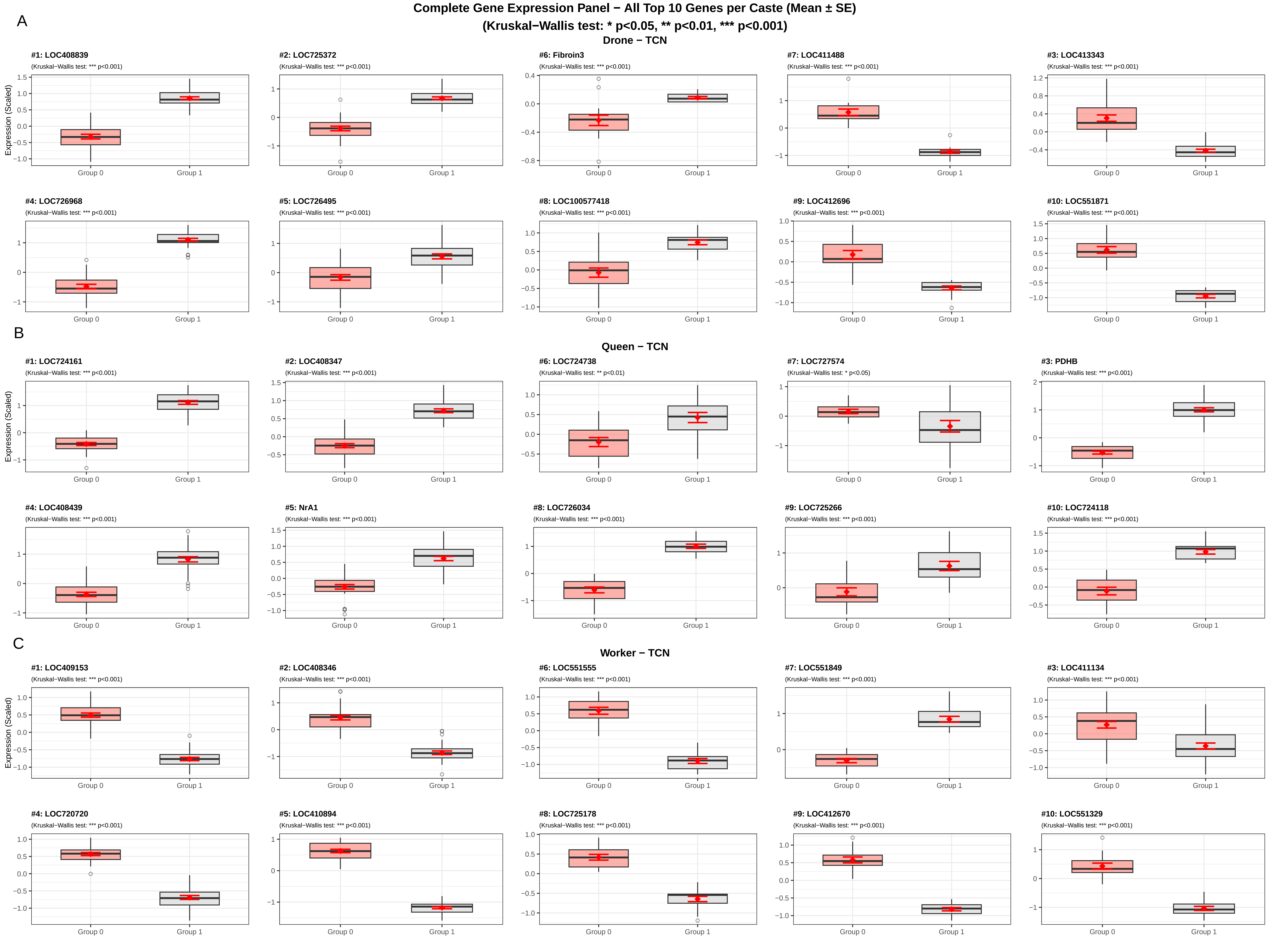
Complete Gene Expression Panel − All Top 10 Genes per Caste (Mean ± SE)
(Kruskal−Wallis test: * p<0.05, ** p<0.01, *** p<0.001)
A
Drone − TCN
#1: LOC408839
(Kruskal−Wallis test: *** p<0.001)
#2: LOC725372
(Kruskal−Wallis test: *** p<0.001)
#6: Fibroin3
(Kruskal−Wallis test: *** p<0.001)
#7: LOC411488
(Kruskal−Wallis test: *** p<0.001)
#3: LOC413343
(Kruskal−Wallis test: *** p<0.001)
#4: LOC726968
(Kruskal−Wallis test: *** p<0.001)
#5: LOC726495
(Kruskal−Wallis test: *** p<0.001)
#8: LOC100577418
(Kruskal−Wallis test: *** p<0.001)
#9: LOC412696
(Kruskal−Wallis test: *** p<0.001)
#10: LOC551871
(Kruskal−Wallis test: *** p<0.001)
B
Queen − TCN
#1: LOC724161
(Kruskal−Wallis test: *** p<0.001)
#2: LOC408347
(Kruskal−Wallis test: *** p<0.001)
#6: LOC724738
(Kruskal−Wallis test: ** p<0.01)
#7: LOC727574
(Kruskal−Wallis test: * p<0.05)
#3: PDHB
(Kruskal−Wallis test: *** p<0.001)
#4: LOC408439
(Kruskal−Wallis test: *** p<0.001)
#5: NrA1
(Kruskal−Wallis test: *** p<0.001)
#8: LOC726034
(Kruskal−Wallis test: *** p<0.001)
#9: LOC725266
(Kruskal−Wallis test: *** p<0.001)
#10: LOC724118
(Kruskal−Wallis test: *** p<0.001)
C
Worker − TCN
#1: LOC409153
(Kruskal−Wallis test: *** p<0.001)
#2: LOC408346
(Kruskal−Wallis test: *** p<0.001)
#6: LOC551555
(Kruskal−Wallis test: *** p<0.001)
#7: LOC551849
(Kruskal−Wallis test: *** p<0.001)
#3: LOC411134
(Kruskal−Wallis test: *** p<0.001)
#4: LOC720720
(Kruskal−Wallis test: *** p<0.001)
#5: LOC410894
(Kruskal−Wallis test: *** p<0.001)
#8: LOC725178
(Kruskal−Wallis test: *** p<0.001)
#9: LOC412670
(Kruskal−Wallis test: *** p<0.001)
#10: LOC551329
(Kruskal−Wallis test: *** p<0.001)
Expression (Scaled)
Group 0
Group 1
