# Supplementary material for: Integrating WGCNA, TCN, and Alternative Splicing to Map Early Caste Programs in Day-2 Honeybee Larvae
Source: Genes (Basel). 2025 Nov 26;16(12):1409. doi: 10.3390/genes16121409 (PMC12733025; doi:10.3390/genes16121409)
Supplement: Supplementary file 1 [file genes-16-01409-s001.zip › Supplemental/Supplemental Figure 3.pdf]

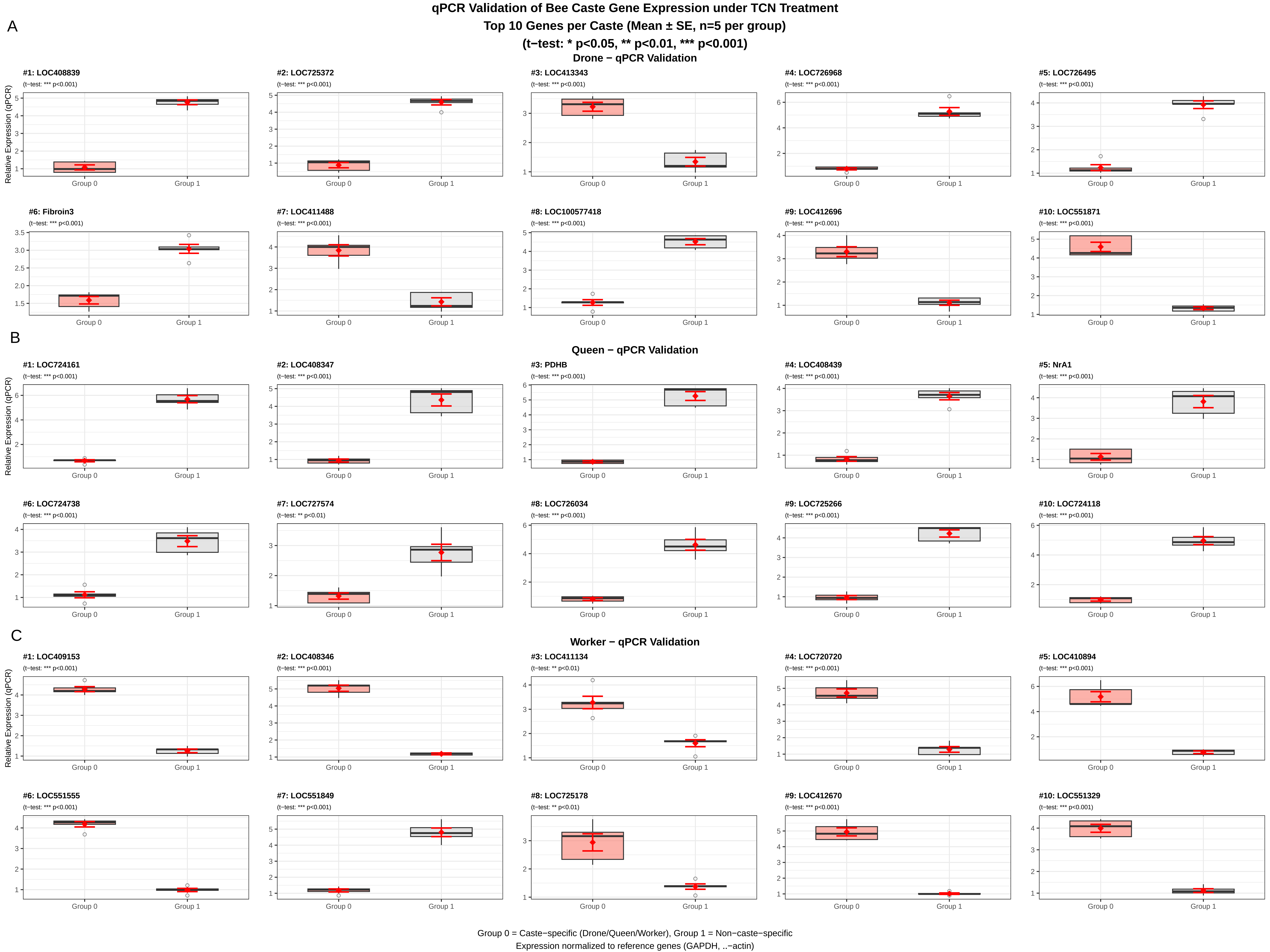
qPCR Validation of Bee Caste Gene Expression under TCN Treatment
Top 10 Genes per Caste (Mean ± SE, n=5 per group)
(t−test: * p<0.05, ** p<0.01, *** p<0.001)
A
Drone − qPCR Validation
Relative Expression (qPCR)
#1: LOC408839
(t−test: *** p<0.001)
#2: LOC725372
(t−test: *** p<0.001)
#3: LOC413343
(t−test: *** p<0.001)
#4: LOC726968
(t−test: *** p<0.001)
#5: LOC726495
(t−test: *** p<0.001)
#6: Fibroin3
(t−test: *** p<0.001)
#7: LOC411488
(t−test: *** p<0.001)
#8: LOC100577418
(t−test: *** p<0.001)
#9: LOC412696
(t−test: *** p<0.001)
#10: LOC551871
(t−test: *** p<0.001)
B
Queen − qPCR Validation
Relative Expression (qPCR)
#1: LOC724161
(t−test: *** p<0.001)
#2: LOC408347
(t−test: *** p<0.001)
#3: PDHB
(t−test: *** p<0.001)
#4: LOC408439
(t−test: *** p<0.001)
#5: NrA1
(t−test: *** p<0.001)
#6: LOC724738
(t−test: *** p<0.001)
#7: LOC727574
(t−test: ** p<0.01)
#8: LOC726034
(t−test: *** p<0.001)
#9: LOC725266
(t−test: *** p<0.001)
#10: LOC724118
(t−test: *** p<0.001)
C
Worker − qPCR Validation
Relative Expression (qPCR)
#1: LOC409153
(t−test: *** p<0.001)
#2: LOC408346
(t−test: *** p<0.001)
#3: LOC411134
(t−test: ** p<0.01)
#4: LOC720720
(t−test: *** p<0.001)
#5: LOC410894
(t−test: *** p<0.001)
#6: LOC551555
(t−test: *** p<0.001)
#7: LOC551849
(t−test: *** p<0.001)
#8: LOC725178
(t−test: ** p<0.01)
#9: LOC412670
(t−test: *** p<0.001)
#10: LOC551329
(t−test: *** p<0.001)
Group 0
Group 1
Group 0 = Caste−specific (Drone/Queen/Worker), Group 1 = Non−caste−specific
Expression normalized to reference genes (GAPDH, ..−actin)
