# Supplementary material for: Integrating WGCNA, TCN, and Alternative Splicing to Map Early Caste Programs in Day-2 Honeybee Larvae
Source: Genes (Basel). 2025 Nov 26;16(12):1409. doi: 10.3390/genes16121409 (PMC12733025; doi:10.3390/genes16121409)
Supplement: Supplementary file 1 [file genes-16-01409-s001.zip › Supplemental/Supplemental Figure 1.pdf]

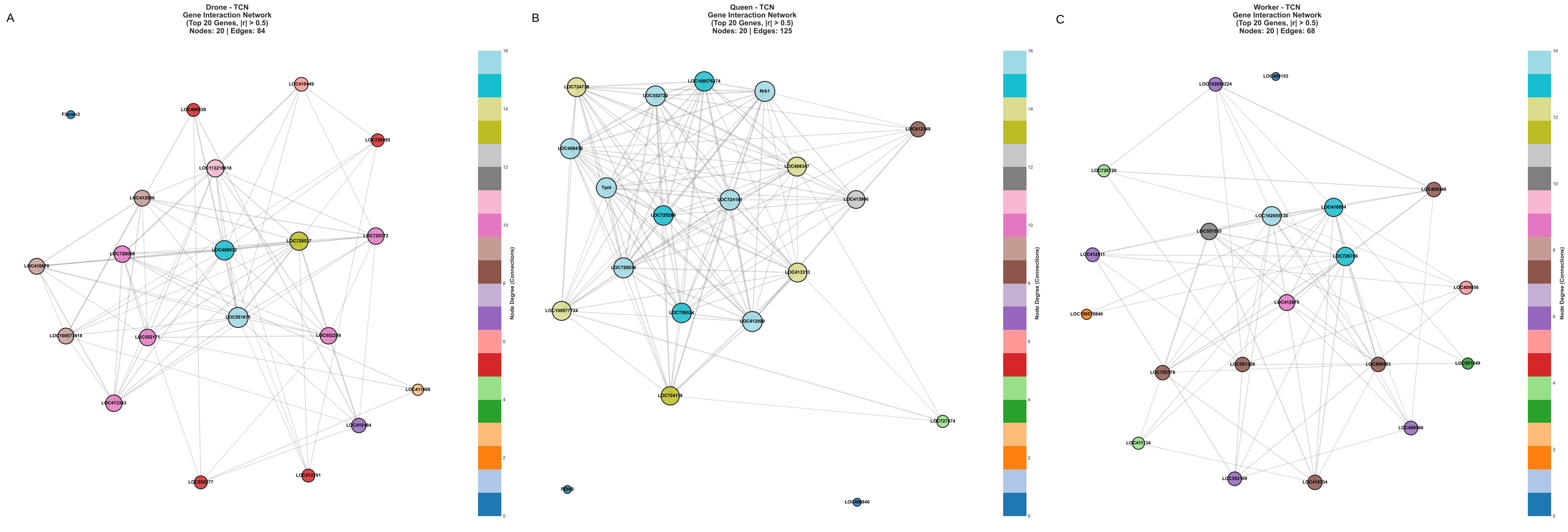
A
Drone - TCN
Gene Interaction Network
(Top 20 Genes, |r| > 0.5)
Nodes: 20 | Edges: 84
Node Degree (Connections)
B
Queen - TCN
Gene Interaction Network
(Top 20 Genes, |r| > 0.5)
Nodes: 20 | Edges: 125
Node Degree (Connections)
C
Worker - TCN
Gene Interaction Network
(Top 20 Genes, |r| > 0.5)
Nodes: 20 | Edges: 68
Node Degree (Connections)
